# Mechanism of Mitochondrial Kinetic Imbalance and Nrf2 Signaling Pathway-Mediated Oxidative Stress in Nickel and/or Chromium-Induced Kidney Injury in Mice

**DOI:** 10.3390/antiox13080980

**Published:** 2024-08-13

**Authors:** Jun Du, Zhengqing Li, Xianhong Cao, Qiurong Qi, Luqi Wang, Ping Liu, Yifei Chen, Guoliang Hu, Xiaoquan Guo, Xiaona Gao

**Affiliations:** 1 Jiangxi Provincial Key Laboratory for Animal Health, Institute of Animal Population Health, College of Animal Science and Technology, Jiangxi Agricultural University, Nanchang 330045, China; dujun180@126.com (J.D.); lizhengqing@stu.njau.edu.cn (Z.L.); xhcao02123@163.com (X.C.); tdcqwlkq@163.com (Q.Q.); 15063227626@163.com (L.W.); pingliujx@163.com (P.L.); chenyfffly@163.com (Y.C.); hgljx3818@jxau.edu.cn (G.H.); xqguo20720@jxau.edu.cn (X.G.); 2 Department of Animal Science, Jiangxi Biological Vocational College, Nanchang 330200, China

**Keywords:** mice, nickel, chromium, kidney, oxidative stress, mitochondrial dynamics

## Abstract

Nickel and chromium are both common heavy metals that pose serious environmental and health hazards. However, the exact mechanism by which nickel and/or chromium cause renal injury is unclear. Therefore, we explored the molecular mechanisms of renal injury caused by nickel and/or chromium poisoning from the perspective of mitochondrial dynamics and the Nrf2 antioxidant pathway. In this study, eighty 6-week-old C57BL/6J mice were randomly divided into four groups: control (Con, untreated), nickel (Ni, 110 mg/L Ni^2+^), chromium (Cr, 50 mg/L Cr^6+^), and combined nickel–chromium (Ni + Cr, 110 mg/L Ni^2+^, 50 mg/L Cr^6+^). The results showed that chronic nickel and/or chromium exposure inhibited body weight gain and impaired kidney function and structure in mice. Chronic nickel and/or chromium exposure led to the disruption of mitochondrial dynamics and thus induced oxidative stress. On the other hand, the Nrf2 antioxidant pathway may play an important regulatory role in mitigating oxidative stress-induced oxidative damage in kidney. The present study partially elucidated the molecular mechanism of renal injury induced by nickel and/or chromium exposure in mice and the regulatory role of the Nrf2 pathway in inducing oxidative injury from the perspective of mitochondrial dynamics. This provides a theoretical basis for the development of prevention and control strategies, and environmental protection measures.

## 1. Introduction

Nickel and chromium are important transition metal elements with a wide range of applications in the modern industry. Nickel is commonly used in the production of alloys, the manufacture of nickel–cadmium batteries, catalysts and electroplating [1,2,3]. Chromium has many applications in the chemical and leather industries, as well as in automotive manufacturing and aerospace [4,5]. Ore mining and processing and industrial emissions are the main causes of nickel and chromium pollution, releasing large quantities of nickel compounds and chromium compounds, and these emissions enter and contaminate the external environment in the form of wastewater, exhaust gases and solid waste [6,7,8]. Jinchang City is the largest nickel and copper production base in China and is also known as the “Nickel City” in China. As a result of the exploitation of metal mineral resources, mines and the surrounding environment have been polluted and damaged, leading to serious impacts on biological health [9,10]. China’s Ministry of Ecology and Environment and the Ministry of Land and Resources released the National Soil Pollution Survey Bulletin, which showed that the exceedance rate of chromium in China had reached 1.1 percent. In mining and smelting areas, as well as in major chromium-related industries, soil chromium concentrations can reach hundreds of times the reference screening value (2500 mg/kg), resulting in severe chromium pollution [11,12]. With the increasing emphasis on environmental protection and health, it is increasingly important to explore in depth and comprehensively the mechanisms by which nickel and/or chromium promote organism damage.

The kidney is the most important organ for the body’s metabolism, as all kinds of toxic substances in the body are mainly metabolized by the kidneys. At the same time, these poisonous substances will inevitably cause damage to the kidneys [13,14,15,16]. The kidneys are the main target organ for nickel, and prolonged exposure to nickel can cause nickel to accumulate in the kidneys, leading to severe kidney damage [17]. Exposure to nickel causes renal dysfunction, induces pathological changes in the kidneys, and can induce oxidative stress and inflammatory responses leading to apoptosis of renal cells [18]. The toxic effects of Cr^6+^ mainly include damage to various organs and systems in the animal’s body. Additionally, chromium binds more readily to proteins and DNA than many other metal ions and can induce apoptosis and inflammatory responses in kidney cells [19,20,21,22]. However, the mechanism of renal damage by nickel and chromium and their combined exposures has not yet been clarified. In addition to cellular studies, it is crucial to investigate specific pathways and correlations at the molecular level in order to study the renal damage caused by chronic nickel and/or chromium exposure. This will highlight the innovation and significance of the present study.

Renal physiological functions are mainly dependent on mitochondrial function. The resting metabolic rate and mitochondrial content of the kidneys are second only to that of the heart, and there is a close link between disturbances in mitochondrial dynamics and renal injury [23]. Mitochondria are constantly dividing and fusing, forming a highly complex mitochondrial network that promotes coordination between mitochondria and other organelles [24]. Among them, mitochondrial division is mainly regulated by dynamin-related protein 1 (Drp1), and the process of mitochondrial fusion mainly involves mitofusin 2 (Mfn2) and optic atrophy 1 (OPA1), which work synergistically to ensure the repair of damaged mitochondria [25,26,27,28]. PGC-1alpha (PGC-1α) is an important regulator that affects mitochondrial production, energy metabolism and cellular respiration [29]. Sirtuin 1 (Sirt1), which is closely related to PGC-1α, enhances its regulatory function by activating PGC-1α, and the synergistic action of PGC-1α and Sirt1 is essential for maintaining healthy intracellular mitochondria, especially in organs with active energy metabolism [30]. In response to oxidative damage, the body activates its antioxidant defense system. The transcription factor nuclear factor erythroid-derived 2-like 2 (Nrf2) is a key component of the cellular antioxidant system and is highly expressed in the kidney. Nrf2 promotes intracellular free radical scavenging by regulating the transcriptional activity of antioxidant- and detoxification-related genes in tissues, thereby counteracting and mitigating cellular damage caused by oxidative stress [31,32]. Studying the mechanisms regulating mitochondrial dynamics is essential for understanding cellular metabolism, mechanisms of renal disease generation and for therapeutic and preventive purposes. The Nrf2 signaling pathway plays an important role in attenuating oxidative damage to cells. However, studies on the specific mechanisms by which nickel and/or chromium cause mitochondrial kinetic imbalances in the mouse kidney and on the body’s resistance to oxidative damage have been rarely reported.

Currently, most of the studies on renal injury from nickel and/or chromium exposure are limited to the cellular level and mostly involve acute staining experiments. There are very few studies on the specific molecular mechanisms of renal injury caused by chronic nickel and/or chromium exposure. The aim of this study was to investigate the relationship between oxidative damage in the mouse kidney caused by chronic nickel and/or chromium exposure and mitochondrial kinetic homeostasis from the perspective of mitochondrial dynamics, as well as the molecular mechanisms by which the Nrf2 signaling pathway plays a role in oxidative damage in the kidney. This study provides a more in-depth and comprehensive theoretical reference for understanding renal injury caused by chronic nickel and/or chromium exposure.

## 2. Materials and Methods

### 2.1. Animals and Experimental Design

Eighty 4-week-old SPF-grade healthy female C57BL/6J mice with no significant differences in body weight were purchased from Changsha Tianqin Biotechnology Co. (Changsha, China). The mice were purchased from Changsha Tianqin Biotechnology Co., Ltd., (Changsha, China), license number SCXK(Xiang)2019-0014. The mice were purchased and housed normally for 14 days to alleviate transportation and other stresses. After 14 days, the mice were randomly divided into four groups of 20 mice each: the Con group, the Ni group, the Cr group, and the Ni + Cr group. The Con group drank ultrapure water, the Ni group drank water containing 110 mg/L Ni^2+^, the Cr group drank water containing 50 mg/L Cr^6+^, and the Ni + Cr group drank water containing 110 mg/L Ni^2+^ and 50 mg/L Cr^6+^. The source of Ni^2+^ was NiCl_2_·6H_2_O and the source of Cr^6+^ was K_2_Cr_2_O_7_. The experimental period was 16 weeks, during which all groups had free access to water and were fed ordinary SPF-grade vacuum-packed mouse chow (Beijing Keao Xieli Feed Co., Ltd., Beijing, China). Animal experimental procedures were conducted in strict accordance with the regulations of the Ethics Committee for Physical Experiments (Nanchang University, Nanchang, China) under the license number Approval NO. NCULAE-20220928008. The Cr^6+^ dose in this study was referenced from Thompson et al. and the Ni^2+^ dose was referenced from Saini et al. [33,34].

### 2.2. Sample Collection and Administration

Sampling was performed at the end of week 16, and water and food were withheld for 12 h prior to sampling. After we euthanized the mice using isoflurane gas, we pressed the eyeballs of the mice to make them protrude as much as possible, removed the eyeballs quickly with forceps and collected the blood. After the blood was allowed to stand at room temperature for 40 min, it was centrifuged at 4 °C and 3000 r/min for 15 min and the supernatant was collected and placed in an ultra-low temperature refrigerator at −80 °C for storage. After weighing the kidneys, a portion of the sample was placed in an RNA preservation solution (Seville Biotechnology Co., Ltd., Wuhan, China) for subsequent qRT-PCR experiments. A portion of the samples was placed in 2.5% glutaraldehyde fixative (Guanyin Biotechnology Co., Ltd., Nanchang, China) for subsequent ultrastructural observation experiments. A portion of the samples were preserved directly in an ultra-low temperature refrigerator (Haier Company, Qingdao, China) for subsequent determination of antioxidant enzymes, renal function and protein immunoblotting experiments. A portion of the samples was placed in a 4% paraformaldehyde solution (Boster Biotechnology Co., Ltd., Wuhan, China) for subsequent pathology section observation experiments. We followed animal protection regulations and ethical guidelines to ensure that standard experimental procedures were performed on the mice and that samples were strictly protected from any contamination, thus ensuring the accuracy and reliability of the study results.

### 2.3. Observation of Kidney Microstructure

The cleaned kidney tissue samples were fixed with 4% paraformaldehyde for 48 h, then soaked in graded concentrations of alcohol, transparentized in xylene, soaked in paraffin, cut into 5 μm-thick sections and stained with hematoxylin and eosin (Solarbio Science & Technology Co., Ltd., Beijing, China). Scans were performed using a panoramic quantitative tissue cell analyzer (TissueGnostics Company, Vienna, Austria) and observed and analyzed using the Tissue FAXS Viewer software 7.0.

### 2.4. Ultrastructural Observation of the Kidney

Kidney samples were cut into 1.0 mm^3^ pieces and fixed in a 2.5% glutaraldehyde solution. After rinsing with 0.1 M PBS buffer (pH = 7.4), the samples were fixed with a 1% osmium solution for 2 h. Subsequent steps included gradient ethanol dehydration, embedding in Epon 812 epoxy resin embedding agent (Hyde Entrepreneurship Biotechnology Co., Ltd., Beijing, China), sectioning and double staining. The samples were observed using an HT7700 transmission electron microscope (Hitachi, Tokyo, Japan) to examine the ultrastructure of renal tissues.

### 2.5. Measurement of Kidney Function

We used the kits provided by the Nanjing Jiancheng Bioengineering Institute (Nanjing, China) to detect the levels of creatinine (Cat. No: C011-2-1) and urea nitrogen (Cat. No: C013-2-1) in the serum, strictly following the operating procedures outlined in the kit instructions.

### 2.6. Determination of Antioxidant Indicators

We used the kit provided by the Nanjing Jiancheng Bioengineering Institute (Nanjing, China) to detect the levels of malondialdehyde (Cat. No: A003-1-2), catalase (Cat. No: A007-1-1) and total antioxidant capacity (Cat. No: A015-1-2) in the mouse serum. Measurements and experiments were performed in strict accordance with the procedures outlined in the kit manual.

### 2.7. Quantitative Real-Time Polymerase Chain Reaction (qRT-PCR)

Total kidney RNA was extracted according to the instructions of the TransZol Up Plus RNA Kit (Takara Bio, Kusatsu, Shiga, Japan, Cat. No: 9109). The extracted total RNA was reverse transcribed to synthesize cDNA, following the instructions provided by the reverse transcription kit (TransGen Biotech Co., Ltd., Beijing, China, Cat. No: AU341-02). Real-time qPCR reactions were carried out using cDNA as a template and the amplification system according to the instruction manual of the qRT-PCR kit (Vazyme Biotech Co., Ltd., Nanjing, China, Cat. No: Q141-02). The instrument used for amplification reactions was Quant StudioTM7, which detects changes in the mRNA expression of relevant genes. Table 1 lists the primers used for qPCR. The measured CT values were calculated using the 2^−ΔΔCT^ statistical method with β-actin as an internal reference.

### 2.8. Western Blot Analysis

Total protein from mouse kidney tissue was extracted according to the instructions of the RIPA lysate kit (Solarbio Science & Technology Co., Ltd., Beijing, China, Cat. No: R0010). The concentration of the extracted protein was determined using a BCA protein concentration assay kit (Solarbio Science & Technology Co. Technology Co., Ltd., Beijing, China, Cat. No: PC0020). SDS-PAGE gel electrophoresis was then performed, and the proteins were transferred to a PVDF membrane. The membrane was incubated with primary antibody at 4 °C overnight. After incubation with horseradish peroxidase-labeled secondary antibody for 1 h, the PVDF membrane was immersed in prepared ECL chemiluminescence developer (ECL, Abbkine Scientific Co., Ltd., Wuhan, China, Cat. No: BMU101) for 30 s and then developed with a Bio-Rad ChemiDoc Touch protein imager (Bio-Rad, Des Plaines, IL, USA). The primary antibodies, Nrf2 (Cat. No: WL02135), Keap1 (Cat. No: WL03285), HO-1 (Cat. No: WL02400), NQO1 (Cat. No: WL04860), Drp1 (Cat. No: WL03028), Sirt1 (Cat. No: WL02995), GAPDH (Cat. No: WL01114) and PGC-1α (Cat. No: WL02123), were purchased from Wanleibio Company (Shenyang, China); the primary antibodies, Mfn2 (Cat. No: sc-515647) and OPA1 (Cat. No: sc-393296), were purchased from Santa Cruz Biotechnology Inc. (Santa Cruz, CA, USA). Secondary antibodies were purchased from Proteintech Group, Inc. (Wuhan, China). The dilution ratio of the antibody was strictly in accordance with the recommended dilution of the purchased product.

### 2.9. Statistical Analysis

The data were summarized using WPS Office and analyzed using one-way analysis of variance (ANOVA) and least significant difference (LSD) tests via SPSS 17.0 (SPSS Inc., Chicago, IL, USA). Statistical results are presented as mean ± standard error and the graphical results were plotted using GraphPad Prism 6.01 (GraphPad Inc., La Jolla, CA, USA). *p* > 0.05 indicates a non-significant difference, *p* < 0.05 indicates a significant difference and *p* < 0.01 indicates a highly significant difference.

## 3. Results

### 3.1. Impairments of Renal Function in Mice by Nickel and/or Chromium

**Figure 1:**
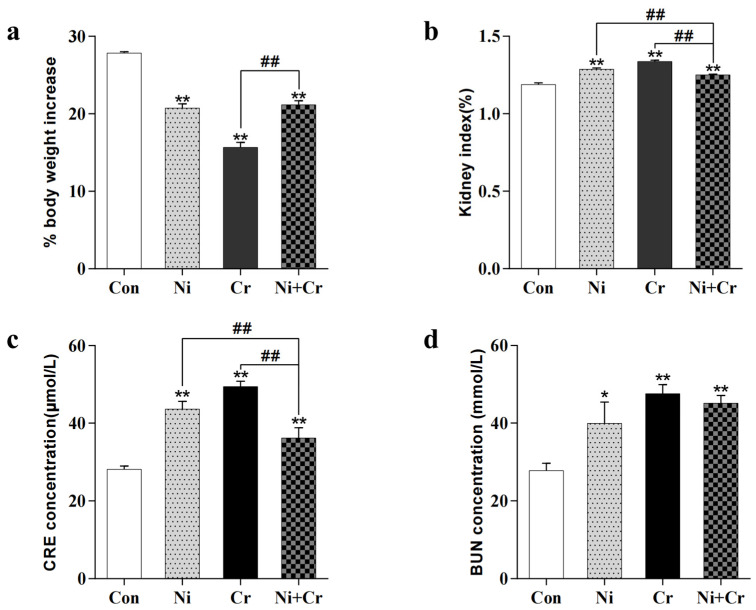
(**a**) Change in body weight growth rate. (**b**) Change in renal coefficient. (**c**) Change in serum CRE concentration. (**d**) Change in serum BUN concentration. Note: the symbol “*” indicates significant differences compared to the control group (* *p* < 0.05, ** *p* < 0.01); the symbol “^#^” indicates significant differences between the corresponding experimental groups (^##^ *p* < 0.01).

Body weight growth rates are shown in Figure 1a. Each exposure group showed an extremely significant decrease in body weight growth rate compared to the control group (*p* < 0.01). The Ni + Cr group showed a significant increase in body weight growth rate compared to the Cr group (*p* < 0.01). Renal coefficients, as shown in Figure 1b, were significantly higher (*p* < 0.01) in all exposed groups of mice compared to the Con group, and showed a significant decreasing trend (*p* < 0.01) in the Ni + Cr group of mice compared to the Ni and Cr groups. Renal function results are shown in Figure 1c,d. Serum creatinine (CRE) and urea nitrogen (BUN) levels were significantly increased in all exposed groups of mice compared to the Con group (*p* < 0.01), and CRE levels were significantly decreased in the Ni + Cr group compared to the Ni group and Cr group (*p* < 0.05 or *p* < 0.01).

### 3.2. Effects of Nickel and/or Chromium Exposure on the Pathologic Histology of the Mouse Kidney

**Figure 2:**
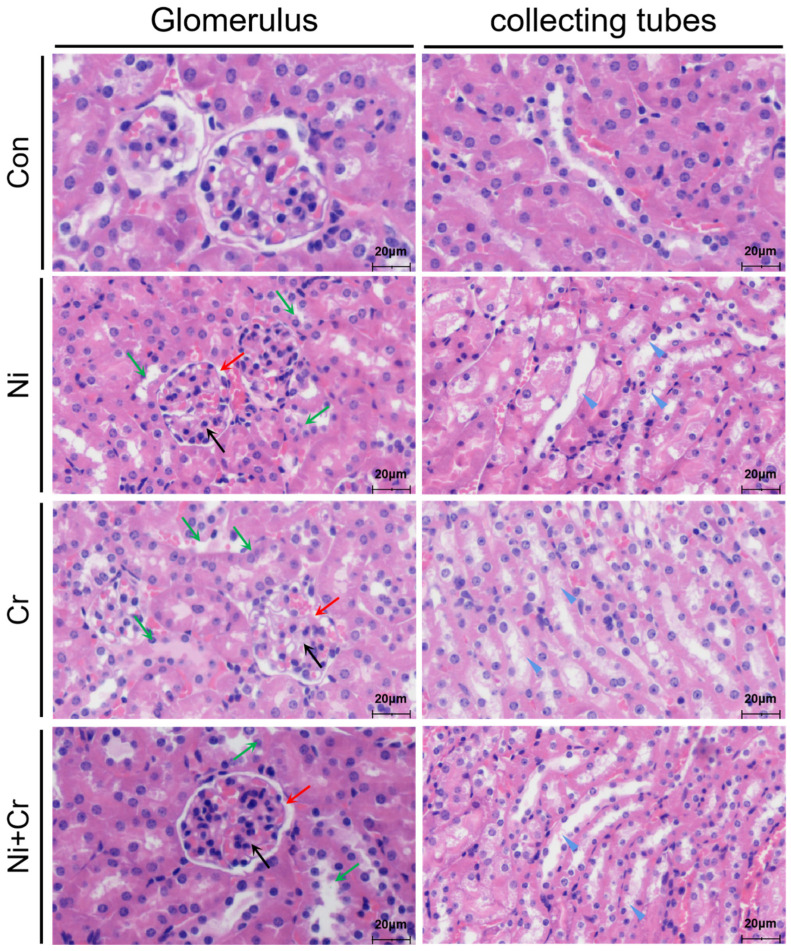
Histology of nickel–chromium on kidney pathology in mice (scale bar: 20 μm). Note: black arrows show glomeruli; red arrows show the narrowing of the lumen of the renal capsule; green arrows show degeneration, necrosis, and shedding of epithelial cells in the lumen of the renal tubule; blue triangles show the shedding of cells and cellular debris in the collecting ducts.

Histopathological sections of mouse kidneys are shown in Figure 2. Kidney tissues of mice in the Con group showed clear and intact cellular structures, intact glomerular structures without agglutination, a regular arrangement of tubular epithelial cells, clear and intact collecting duct structures, intact cell membranes and normal staining of the cell nuclei. In contrast, the renal tissues of mice in the Ni, Cr and Ni + Cr groups showed different degrees of structural damage. The number of glomerular cells increased and aggregated into clusters, with irregular glomerular contours, narrowing of the lumen of some renal capsules and even adhesion to the capsule. Additionally, there was a narrowing of the lumen in renal tubules, accompanied by swelling of the epithelial cells, in which granular, cellular and glassy tubular patterns were observed. At the same time, the epithelial cells of renal tubules were degenerated, ruptured and necrotic, the brush border was detached, and the basement membrane of some renal tubules was exposed, forming a bare membrane. The arrangement of renal tubules and collecting ducts also became irregular.

### 3.3. Effects of Nickel and/or Chromium Exposure on the Ultrastructure of the Mouse Kidney

**Figure 3:**
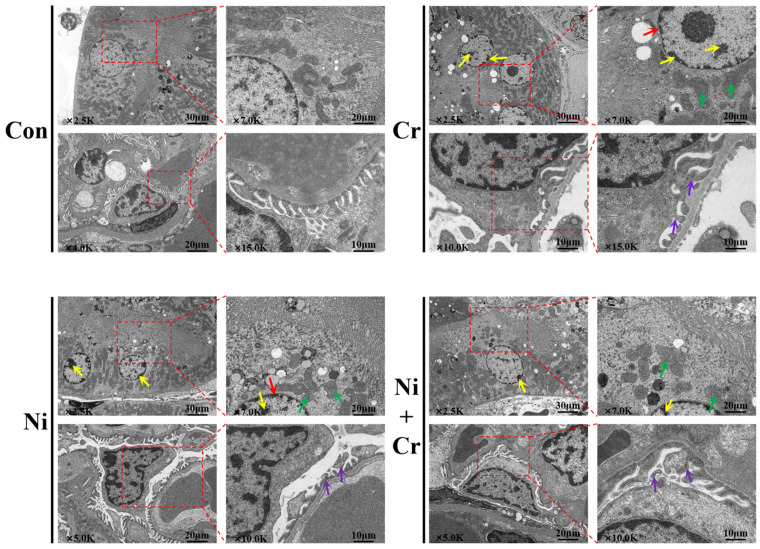
Effects of nickel–chromium on mice kidney tissue ultrastructure. Note: red arrows show nuclear membrane crumpling; yellow arrows show chromatin condensation; green arrows show mitochondrial swelling and distortion of morphology; purple arrows show fusion of peduncle protrusions with curved and swollen morphology. The red dashed box is a partial zoom.

The ultrastructure of mouse renal tissue cells is shown in Figure 3. Renal cells in the Con group were structurally intact, with complete organelle structures and normal mitochondrial morphology, structure and number. The mitochondria of renal tubular epithelial cells were structurally intact, with smooth outer membranes and no swelling. The inner cristae of the mitochondria were arranged in a regular manner. Compared with the Con group, renal cells in all exposed groups showed different degrees of damage, with disorganized renal cell structures, smaller nuclei, atrophied nuclear membranes and chromatin condensed into large dense clusters arranged along the wrinkled nuclear membranes. The mitochondria in renal tubular epithelial cells were swollen, twisted and vacuolated, with a disorganized cristae arrangement. The pedunculated cells had fused peduncles with a curved and swollen morphology.

### 3.4. Effects of Nickel and/or Chromium Exposure on Oxidative Stress in Mice

**Figure 4:**
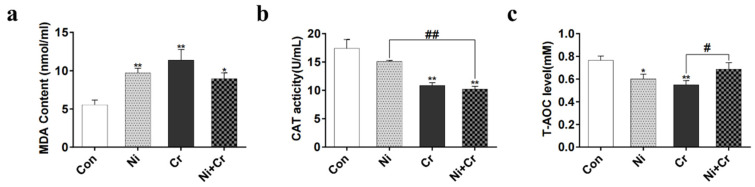
Effects of nickel–chromium on oxidative and antioxidant indices in mice. (**a**) Levels of MDA. (**b**) Activity of CAT. (**c**) Levels of T-AOC. Note: the symbol “*” indicates significant differences compared to the control group (* *p* < 0.05, ** *p* < 0.01); the symbol “^#^” indicates significant differences between the corresponding experimental groups (^#^ *p* < 0.05, ^##^ *p* < 0.01).

The results of oxidative and antioxidant indices are shown in Figure 4a–c. Malondialdehyde (MDA) content was significantly higher in all exposed groups compared to the control group (*p* < 0.05 or *p* < 0.01). Compared with the Con group, the Cr and Ni + Cr groups showed a significant decrease in catalase (CAT) content (*p* < 0.01), and the Ni + Cr group showed a significant decrease in CAT content compared with the Ni group (*p* < 0.01). Compared with the Con group, total antioxidant capacity (T-AOC) activity was significantly lower in the Ni group and Cr group (*p* < 0.05 or *p* < 0.01) and significantly higher in the Ni + Cr group compared with the Cr group (*p* < 0.05).

### 3.5. Effects of Nickel and/or Chromium Exposure on the Relative Expression of Genes and Proteins Associated with Mitochondrial Dynamics

**Figure 5:**
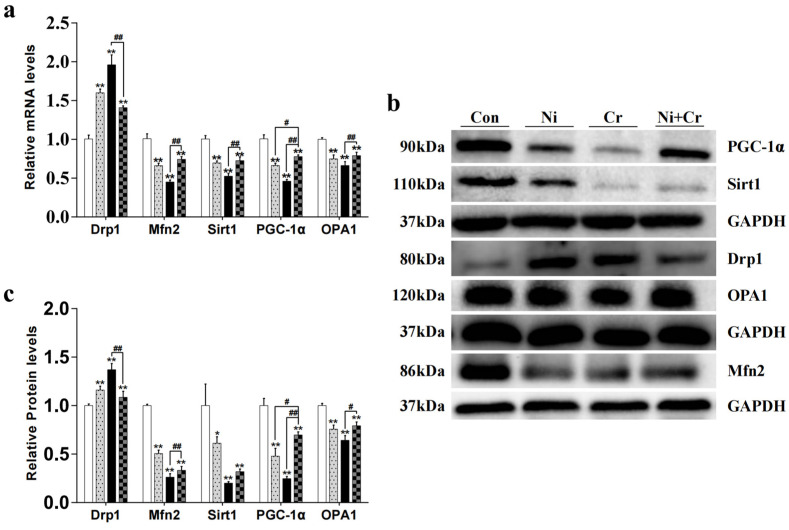
Effects of nickel-chromium on genes and proteins related to mitochondrial dynamics in mice. (**a**) Relative mRNA levels of Drp1, Mfn2, Sirt1, PGC-1α and OPA1. (**b**) Representative western blot images of Drp1, Mfn2, Sirt1, PGC-1α and OPA1. (**c**) Relative protein levels of Drp1, Mfn2, Sirt1, PGC-1α and OPA1. Note: The symbol “*” indicates significant differences compared to the control group (* *p* < 0.05, ** *p* < 0.01). The symbol “^#^” indicates significant differences between the corresponding experimental groups (^#^ *p* < 0.05, ^##^ *p* < 0.01).

The effects of nickel and/or chromium exposure on the relative expression of genes and proteins related to mitochondrial dynamics are shown in Figure 5. Compared with the Con group, the mRNA and protein expression levels of Drp1 in all exposed groups showed a significant increase (*p* < 0.01). The mRNA and protein expression levels of Drp1 in the Ni + Cr group showed a significant decrease compared with those in the Cr group (*p* < 0.01). Compared with the Con group, the mRNA and protein expression levels of Mfn2 in all exposure groups showed a significant decreasing trend (*p* < 0.01). In the Ni + Cr group compared with the Cr group, the mRNA and protein expression levels of Mfn2 showed a significant increasing trend (*p* < 0.01). Compared with the Con group, the mRNA expression levels and protein expression levels of Sirt1 in all exposed groups showed a significant decreasing trend (*p* < 0.05 or *p* < 0.01). The mRNA expression levels of Sirt1 in the Ni + Cr group were significantly increased compared with that in the Cr group (*p* < 0.01). Compared with the Con group, the mRNA expression levels and protein expression levels of PGC-1α were significantly decreased in all exposed groups (*p* < 0.01). The mRNA expression levels and protein expression levels of PGC-1α in the Ni + Cr group were significantly increased compared with those in the Ni and Cr groups (*p* < 0.05 or *p* < 0.01). Compared with the Con group, the mRNA expression levels and protein expression levels of OPA1 in all exposed groups showed a significant decreasing trend (*p* < 0.01). The mRNA expression levels and protein expression levels of OPA1 in the Ni + Cr group showed a significant increasing trend compared with that in the Cr group (*p* < 0.05 or *p* < 0.01).

### 3.6. Effects of Nickel and/or Chromium Exposure on the Relative Expression of Key Molecular Genes and Proteins of the Nrf2 Signaling Pathway

**Figure 6:**
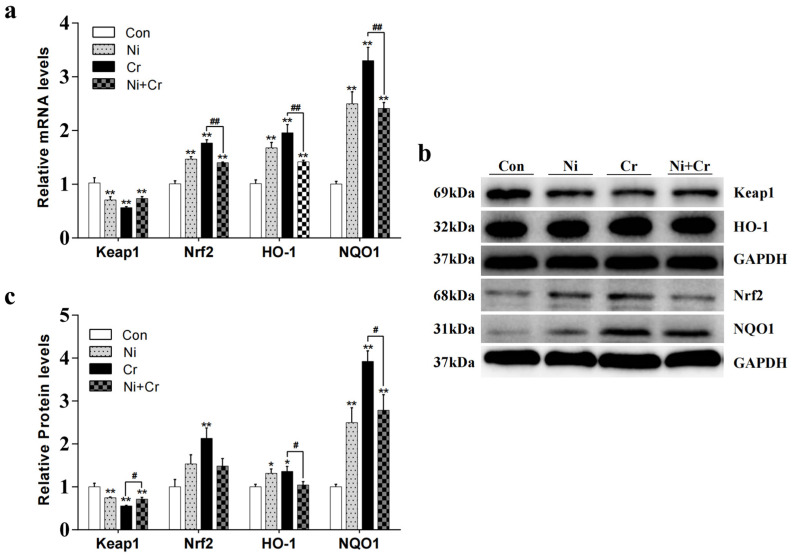
Effects of nickel–chromium on genes and proteins related to Nrf2 pathway in mice. (**a**) Relative mRNA levels of Keap1, Nrf2, HO-1 and NQO1. (**b**) Representative Western blot images of Keap1, Nrf2, HO-1 and NQO1. (**c**) Relative protein levels of Keap1, Nrf2, HO-1 and NQO1. Note: the symbol “*” indicates significant differences compared to the control group (* *p* < 0.05, ** *p* < 0.01); the symbol “^#^” indicates significant differences between the corresponding experimental groups (^#^ *p* < 0.05, ^##^ *p* < 0.01).

Figure 6 illustrates the effects of nickel and/or chromium exposure on the relative expression levels of genes and proteins associated with key molecules of the Nrf2 signaling pathway. Compared with the Con group, Keap1 mRNA and protein expression levels were significantly lower in all exposed groups (*p* < 0.01); Keap1 protein expression levels were significantly higher in the Ni + Cr group compared with the Cr group (*p* < 0.05). The Nrf2 mRNA expression levels were significantly higher in all exposed groups, and the Nrf2 protein expression levels were significantly higher in the Cr group compared to the Con group (*p* < 0.01). Additionally, the Ni + Cr group showed a significant decrease in Nrf2 mRNA expression levels compared to the Cr group (*p* < 0.01). Compared with the Con group, the HO-1 mRNA expression levels of all exposed groups as well as the HO-1 protein expression levels of the Ni and Cr groups showed a significant upward trend (*p* < 0.05 or *p* < 0.01), and the Ni + Cr group showed a significant decrease in the mRNA and protein expression levels of HO-1 compared with the Cr group (*p* < 0.05). Compared with the Con group, the NQO1 mRNA and protein expression levels in all exposed groups showed a significant increasing trend (*p* < 0.01), and the Ni + Cr group showed a significant decreasing trend in NQO1 mRNA and protein expression levels compared with the Cr group (*p* < 0.05 or *p* < 0.01).

## 4. Discussion

Nickel, chromium and their compounds are multi-system and multi-organ toxicants, and the kidney, as one of the most important metabolic organs, is inevitably damaged by nickel and/or chromium [35,36]. Along with the development of nickel–chromium-related industries and the large-scale mining of nickel–chromium ores, their impacts on biological health and the environment have become increasingly evident [37]. Prolonged exposure of occupational workers, such as miners, to higher concentrations of nickel and/or chromium can lead to the enrichment of nickel and chromium in the body, and the kidneys, as an important metabolic organ of the body, are likely to be damaged [38]. However, there is a lack of clarity regarding the specific mechanisms of renal injury by nickel and/or chromium; therefore, there is a need to investigate the effects of chronic nickel and/or chromium exposure on oxidative damage in the mouse kidney and its molecular mechanisms. In this study, we constructed a nickel- and/or chromium-induced kidney injury model in mice to investigate the molecular mechanisms of nickel- and/or chromium-induced kidney injury from the perspective of renal mitochondrial dynamics and to assess the influence of the Nrf2 pathway on oxidative damage in the kidney.

The renal index is an important indicator of the degree of kidney damage caused by toxic substances [39,40]. Previous studies on the effects of heavy metals on the kidney have shown that heavy metals such as mercury and cadmium can cause serious damage to the structure and function of the animal kidney [41,42]. In this experiment, the kidney coefficients of mice in all exposed groups were significantly higher than those of the control group, suggesting that the kidneys of mice in the exposed groups in this experiment may have been damaged. In order to visually assess the toxicity of nickel and/or chromium on renal tissues, we used hematoxylin and eosin staining and transmission electron microscopy to observe pathological changes and the degree of structural damage to the renal tissues. The results showed that all exposed groups of mice showed varying degrees of damage to the renal structures, indicating that nickel and/or chromium exposure caused damage to the renal structures of mice. To further investigate the effects of nickel and/or chromium exposure on renal function in mice, we measured the levels of CRE and BUN. Analysis of the results showed that CRE and BUN levels were abnormally elevated in all exposed groups of mice, suggesting that nickel and/or chromium caused serious damage to kidney function in the mice. In this experiment, we demonstrated that nickel and/or chromium caused damage to the structure and function of the mouse kidney by integrating changes in renal coefficients, renal pathohistology, renal cell ultrastructure and renal function. The above results indicate that we have successfully established an animal toxicity model for subchronic exposure to nickel and/or chromium, leading to nephrotoxicity in mice. However, the specific mechanisms by which nickel and chromium exposure leads to renal injury need to be further explored.

Mitochondrial dynamic homeostasis plays an important role in maintaining stable cellular energy production and normal mitochondrial function [43,44]. Mitochondria maintain the normalcy and self-repair of their structure and function through continuous fusion and fission, helping to resist oxidative damage caused by excess ROS in cells [45,46,47]. The fusion and fission of mitochondria in normal cells are in a state of dynamic equilibrium. However, toxic substances, including heavy metals, can disrupt this dynamic equilibrium, causing an imbalance between mitochondrial fission and fusion. This imbalance can lead to cellular damage and ultimately result in oxidative stress and apoptosis [45]. Li et al. showed that HgCl_2_-induced disruption of mitochondrial dynamics activated oxidative stress and led to apoptosis in the mouse kidney [48]. In the present study, ultrastructural analyses showed that nickel/or chromium exposure resulted in mitochondrial swelling, morphological distortion and vacuolization, and the arrangement of cristae within mitochondria was disturbed. At the same time, we detected changes in the mRNA and protein expression levels of factors controlling mitochondrial fusion and division and found that the mRNA and protein expression levels of factors controlling mitochondrial fusion were reduced, while those of factors controlling mitochondrial division were increased. Thus, exposure to nickel and/or chromium leads to aberrant expression of factors associated with the regulation of division and fusion in mouse kidney cells, which leads to an imbalance in mitochondrial dynamics in kidney cells, which in turn induces oxidative damage in the kidney.

Previous studies have shown that the Nrf2-dominated antioxidant pathway plays a key role in resisting oxidative stress and mitochondrial damage and is one of the most important antioxidant mechanisms in vivo [49,50,51]. Other studies have found that Nrf2-mediated antioxidant pathways can play a protective role in a variety of heavy metal poisoning mechanisms [52,53,54]. Heavy metals and toxic substances lead to elevated levels of free radicals in the body, which in turn induce oxidative stress and oxidative damage to kidney cells. In this case, free Nrf2 in the cytoplasm enters the nucleus, combines with antioxidant gene sequences on the ARE, and regulates the expression of downstream antioxidant genes, such as NQO1 and HO-1, which improves the antioxidant capacity of the cell and maintains the dynamic balance of oxidation–antioxidation [55,56]. In this experiment, after exposure to nickel and/or chromium, the expression of Keap1 genes and proteins in mouse kidney tissues was significantly reduced, while the expression levels of Nrf2 and its downstream target genes, HO-1 and NQO1, were significantly increased. This suggests that the Nrf2 pathway plays an important role in nickel- and/or chromium-induced renal injury and is an important antioxidative stress pathway that exerts a protective effect on cells. When cells are stimulated by oxidative stress, Nrf2 gradually translocates from the cytoplasm to the nucleus, activates the expression of various antioxidant genes, such as HO-1 and NQO1, and promotes intracellular antioxidant synthesis to scavenge oxidative free radicals [31,57]. Thus, activation of the Nrf2 pathway has a role in protecting the kidney from oxidative stress injury, whereas nickel- and/or chromium-induced oxidative stress activates the Nrf2 pathway, which is involved in nickel- and/or chromium-induced kidney injury. In this experiment, the antioxidant effect of the Nrf2 antioxidant pathway was enhanced in kidney cells of all exposed groups, but the mouse kidney cells were still subjected to oxidative damage. We analyzed the reason for this experimental result and found that the antioxidant effect of the Nrf2 pathway might alleviate oxidative damage in mouse kidneys to a certain extent. However, in the present experiment, the kidneys of mice exposed to nickel and/or chromium for a prolonged period experienced severe damage, and the Nrf2 antioxidant pathway was not sufficient to completely alleviate the excessive oxidative damage. The Nrf2 antioxidant pathway can mitigate oxidative stress damage by activating the expression of antioxidant genes, but if oxidative stress is too strong, the regulatory capacity of the Nrf2 pathway may be exceeded. In summary, the Nrf2 signaling pathway may modulate nickel- and/or chromium-induced renal injury by regulating oxidative stress, but the present experiments lacked interventions with Nrf2 signaling pathway blockers, and thus further experimental explorations are needed. In addition, the detailed mechanism of interaction between the Nrf2-mediated antioxidant pathway and mitochondrial kinetic imbalance needs to be explored through in vitro experiments.

## 5. Conclusions

In conclusion, exposure to nickel and/or chromium disrupts the dynamic balance between mitochondrial fusion and fission in the mouse kidney, leading to oxidative damage. In addition, activation of the Nrf2 antioxidant pathway is a cellular defense mechanism against nickel and/or chromium-induced oxidative stress. However, despite the protective effects of the Nrf2 pathway, the extent of oxidative damage to renal cells suggests that it has limitations in fully mitigating the adverse effects of chronic exposure to nickel and/or chromium.

## Figures and Tables

**Table 1:** Primer sequences.

Genes	Accession Number	Primers Sequences (5′-3′)
β-Actin	NM_007393.5	F: 5′-GTACTCTGTGTGGATCGGTGG-3′ R: 5′-GCAGCTCAGTAACAGTCCG-3′
Keap1	NM_016679.4	F: 5′-GGCAGGACCAGTTGAACAGT-3′ R: 5′-GGGTCACCTCACTCCAGGTA-3′
Nrf2	NM_010902.5	F: 5′-CAGCCATGACTGATTTAAGCAG-3′ R: 5′-CAGCTGCTTGTTTTCGGTATTA-3′
HO-1	NM_010442.2	F: 5′-AGGTACACATCCAAGCCGAGA-3′ R: 5′-CATCACCAGCTTAAAGCCTTCT-3′
NQO1	NM_008706.5	F: 5′-GGTGAGCTGAAGGACTCGAA-3′ R: 5′-ACCACTGCAATGGGAACTGAA-3′
Drp1	NM_001025947.3	F: 5′-CGTAGTGGGAACTCAGAGCA-3′ R: 5′-TGGACCAGCTGCAGAATAAG-3′
Mfn2	NM_001285920.1	F: 5′-GCATTCTTGTGGTCGGAGGAGTG-3′ R: 5′-TGGTCCAGGTCAGTCGCTCATAG-3′
PGC-1α	NM_001402987.1	F: 5′-GGATATACTTTACGCAGGTCGA-3′ R: 5′-CGTCTGAGTTGGTATCTAGGTC-3′
Sirt1	NM_019812.3	F: 5′-CGCTGTGGCAGATTGTTATTAA-3′ R: 5′-TTGATCTGAAGTCAGGAATCCC-3′
OPA1	NM_001199177.2	F: 5′-CAGCTGGCAGAAGATCTCAAG-3′ R: 5′-CATGAGCAGGATTTTGACACC-3′

## Data Availability

The data presented in this study are available in the article.
